# Interferon-induced protein with tetratricopeptide repeats 1 (IFIT1) accelerates osteoclast formation by regulating signal transducer and activator of transcription 3 (STAT3) signalling

**DOI:** 10.1080/21655979.2021.2024333

**Published:** 2022-01-16

**Authors:** Yuanliang Xue, Chuanliang Zhao, Tao Liu

**Affiliations:** a Department of Orthopedics, Clinical Medical College of Shandong University of Traditional Chinese Medicine, Jinan, Shandong, China; b Department of Radiology, Laoling People’s Hospital, Dezhou, Shandong, China; c Department of Pediatric Surgery, Dezhou People’s Hospital of Shandong, Dezhou, Shandong, China

**Keywords:** IFIT1, osteoclast formation, STAT3 signaling, RANKL

## Abstract

Osteoclasts (OCs), the main cause of bone resorption irregularities, may ultimately cause various bone diseases, including osteoarthritis. The objective of this study was to investigate the effect of interferon-induced protein with tetratricopeptide repeats 1 (IFIT1) on OC formation induced by receptor activator of nuclear factor κB (NF-κB) ligand (RANKL) and to further explore its underlying mechanism. IFIT1 expression in Raw264.7 cells treated with macrophage colony-stimulating factor (M-CSF) and RANKL was determined by qRT-PCR. OC formation was detected using tartrate-resistant acid phosphatase (TRAP) staining. The effect of IFIT1 on STAT3 activation was detected using Western blotting. Additionally, Western blotting was used to measure the change in the expression of OC-specific proteins. IFIT1 was highly expressed in Raw264.7 cells after stimulation with M-CSF and RANKL. IFIT1 overexpression accelerated the formation of OCs, as evidenced by the increased number and size of multinuclear cells, and the upregulation of OC-specific proteins, and activated the STAT3 pathway, by inducing phosphorylation of JAK1 and STAT3. However, silencing of IFIT1 inhibited the formation of OCs and a STAT3 inhibitor Stattic weakened the effects of IFIT1. In conclusion, IFIT1 accelerates the formation of OCs, which is caused by RANKL by STAT3 pathway regulation. This study provides a potential basis for further research and for development of drugs for treating bone resorption-related diseases.

## Graphical abstract

**Figure:**
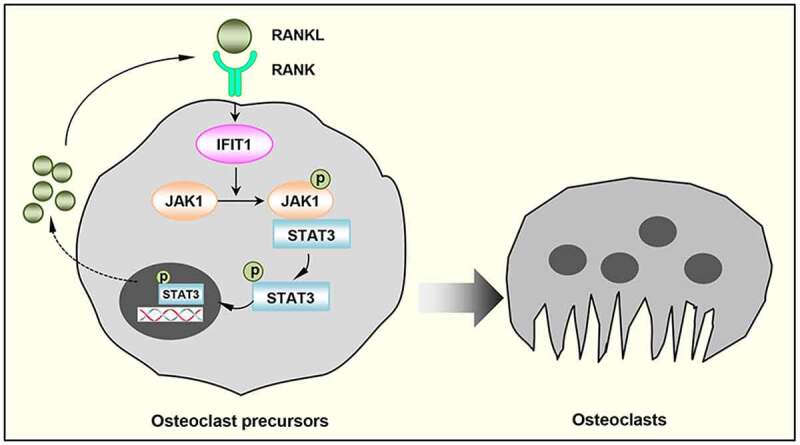

## Introduction

Osteoclasts (OCs) are derived from bone marrow hematopoietic stem cells, and are multinucleated giant cells formed by fusion and highly differentiated OC precursor cells [1]. OCs are the only cell type in mammals with bone resorption activity *in vivo* [2]. The dynamic balance between bone resorption and bone formation plays a critical role in the process of bone remodeling in the body [3]. Changes in the number or activity of OCs can cause an imbalance in bone metabolism, leading to bone diseases such as osteoporosis and osteoarthritis [4,5]. Two important cytokines involved in the differentiation and maturation of OCs are macrophage colony-stimulating factor (M-CSF) and receptor activator of nuclear factor κB (NF-κB) ligand (RANKL) [6,7] Exploring the regulatory mechanisms of OC differentiation and function and discovering novel drug targets can provide a potential basis for improving drug therapy of bone diseases caused by abnormal OCs.

In the event of OC differentiation, RANKL binds to its specific receptor RANK on the precursor cell surface and activates nuclear factor of activated T cell cytoplasmic 1 (NFATc1) and c-Fos, which further increases the expression of OC-specific mediators, including DC-specific transmembrane protein (DC-STAMP) [8], v-ATPase V0 subunit d2 (ATP6V0D2) [9], cathepsin K (CTSK) [10], matrix metalloproteinase-9 (MMP-9) [11], ATPase H+ transporting vacuolar proton pump member I (ATP6i) [12], and tartrate-resistant acid phosphatase (TRAP) [13]. These mediators are OC differentiation biomarkers and are essential for cell–cell fusion, differentiation, bone matrix proteolysis, and bone resorption functions of OCs.

Interferon-induced protein with tetratricopeptide repeats 1 (IFIT1) is a member of the IFIT family which is located on chromosome 10q23.31 and contains two exons. Generally, IFIT1 is expressed at low levels in most cells in the absence of stimuli. However, in the presence of stimuli, such as pathogens, IFIT1 production is increased by secreted interferon, and it is involved in cellular innate immune responses [14]. The biological function of IFIT1 in viral pathogenesis is well established, and inhibition of IFIT1 related immune evasion is considered a critical antiviral strategy [15,16]. Recently, its properties beyond antiviral effects have been demonstrated. For example, IFIT1 was found to be expressed highly in head and neck squamous cell carcinomas and is associated with poor prognoses [17]. Additionally, IFIT1 regulates tumor cell growth and metastasis in oral squamous cell carcinoma [18]. Furthermore, IFIT1 promotes the expression of pro-inflammatory cytokines induced by lipopolysaccharide in human umbilical vein endothelial cells, indicating that IFIT1 is a key participant in the development of atherosclerosis [19]. However, the role of IFIT1 in osteoarthritis and OC formation has not been previously studied.

Signal transducer and activator of transcription 3 (STAT3) is a main member of the STAT family, and its activation leads to the significant production of various cytokines and growth factors to regulate immune response [20]. STAT3 is also involved in the regulation of cell growth, differentiation, death, and tumorigenesis [21,22]. It was reported that, inhibition of STAT3 activation was effective in controlling osteoclast differentiation and formation [23]. Additionally, STAT3 signaling offers a protective function in mice against interleukin (IL)-6 induced osteoarthritis [24]. In this study, changes in IFIT1 expression and the role and pathway of IFIT1 on OC formation were investigated, to provide a novel potential basis for drug therapy of bone diseases caused by abnormal OCs.

## Materials and methods

### Bioinformatic analysis

By searching the Gene Expression Omnibus (GEO) database (http://www.ncbi.nlm.nih.gov/geo), GSE51588, GSE1919, and GSE75181 datasets were screened for targeted chip research. The upregulated genes were analyzed using the ‘limma’ package. The GSE51588 dataset was generated from the knee lateral and medial tibial plateaus of 20 osteoarthritis patients and 5 non-osteoarthritis individuals. In the GSE1919 dataset, we selected GSM34393-GSM34397 as the osteoarthritis samples, while GSM34379, GSM34383, GSM34385, GSM34388, and GSM34391 served as the normal samples. For the GSE75181 dataset, cartilage specimens were obtained from 12 patients with knee osteoarthritis. Primary human chondrocytes were collected and treated with or without human IL-1β. Microarray gene expression profiling was performed between IL-1β-treated and control groups. Gene ontology annotation and KEGG pathway enrichment analyses were conducted using the Clusterprofiler package in R language to investigate the potential signaling pathways.

### Raw264.7 precursor cell culture

Raw264.7 cells (Chinese Academy of Sciences Cell Bank, Shanghai, China) were cultured in Dulbecco’s Modified Eagle’s Medium (Invitrogen, USA) containing 10% fetal bovine serum (HyClone, USA) and 1% streptomycin and penicillin (HyClone, USA) in an incubator at 37°C with 5% CO_2_ and maximum humidity.

### Drug treatment

Raw264.7 cells were seeded into 48-well plates (5 × 10^3^/cm^2^). Cells were then treated with 0, 1, 5, 10, 50, and 100 ng/mL of RANKL (Sigma-Aldrich, USA) for 0–12 days in the presence of 50 ng/mL M-CSF (Sigma-Aldrich, USA).

### Cells transfection

The IFIT1 overexpression plasmid (PCMV6-IFIT1) and control cloning vectors were purchased from OriGene (Rockville, MD, USA). Small interfering RNAs (siRNAs) specifically targeting IFIT1 (si-IFIT1), and scrambled negative control siRNAs were synthesized and purified by RiboBio (Guangzhou, China). Transfection was performed using Lipofectamine 2000 (Invitrogen, Carlsbad, CA, USA) according to the manufacturer’s instructions.

### TRAP staining

TRAP staining was performed according to the instructions of the TRAP staining kit (PMC-AK04F-COS, Whatman, USA). In brief, cells were washed with phosphate buffer saline (PBS), and fixed with TRAP fixative at 4°C for 30 s to 3 min. Subsequently, the cells were washed with PBS and incubated with TRAP solution at 37°C for 45–60 min. Finally, the cells were washed with water, re-stained with hematoxylin for 3 min and observed at microscope. TRAP-positive cells with three or more nuclei were regarded as multinuclear OCs. The OC sizes were quantified using Image-Pro Plus software (version 6.0; Media Cybernetics, USA).

### Real-time quantitative RT-PCR

Total RNA content was isolated from the cells by homogenization using TRIzol (Invitrogen, Carlsbad, CA, USA). The FOTODYNE gel imaging analysis system (Fotodyne, Inc., Hartland, WI, USA) was used to determine the RNA quality and the 28S/18S ratio. cDNA synthesis was performed using a cDNA cycle kit (Thermo Fisher Scientific, Waltham, MA, USA). The primer sequences used for quantitative real-time PCR amplification were 5′-TTTACAGCAACCATGGGAGAGAA-3′ (forward), 5′-CTACGCGATGTTTCCTACGG-3′ (reverse) for IFIT1 and 5′-CCAGCCTTCCTTCTTGGGTAT-3′ (forward), 5′-GGGTGTAAAACGCAGCTCA G-3′ (reverse) for β-actin. The expression level of β-actin was used to normalize the mRNA expression, and the fold change = 2-^ΔΔ^CT was used for calculation.

### Western blot

The proteins were extracted using RIPA Lysis Solution (P0013 C, Beyotime, Shanghai, China) and protein concentrations were measured using a bicinchoninic acid protein assay kit (KeyGen Biotech Co., Ltd, Nanjing, China). The proteins were separated by SDS-PAGE and transferred onto polyvinylidene fluoride membranes (Roche, Switzerland). The membranes were incubated with the following primary antibodies: IFIT1 (ab236256, Abcam, USA), ATP6V0D2 (H00245972-M01A, Abnova, Shanghai, China), DC-STAMP (ab238151, Abcam), ATP6i (H00000525-M02, Abnova), CTSK (H00001513-M01, Abnova), TRAP (ab2721, Abcam), p-JAK1 (ab138005, Abcam), JAK1 (ab133666, Abcam), p-STAT3 (ab267373, Abcam), STAT3 (ab68153, Abcam), c-Fos (ab222699, Abcam), MMP9 (ab76003, Abcam), NFATc1 (GTX09510, GeneTex, USA), and β-actin (C1313, Applygen, Beijing, China). After a 12 h incubation, the membranes were incubated with the secondary antibody, and the intensity of protein expression was detected by ChemiScope 3300 Mini (CLINX, Shanghai, China) using enhanced chemiluminescence (Beyotime, Beijing, China).

### Statistical analysis

All experiments were performed in triplicate and data were presented as the mean ± standard deviation. Data were analyzed using SPSS 19.0 (Chicago, IL, USA). One-way analysis of variance (ANOVA) was used to assess the differences between the groups. Statistical significance was set at *P* < 0.05.

## Results

### IFIT1 was highly expressed in OCs

**Figure 1.**
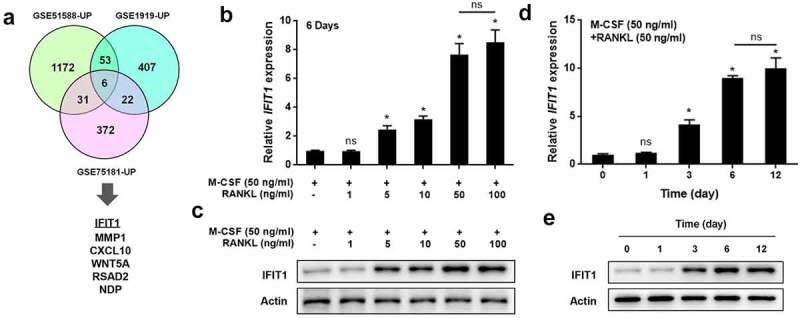
IFIT1 was highly expressed in OCs. (a) Six overlapping genes, including *IFIT1*, were found to be upregulated in the GSE51588, GSE1919, and GSE75181 datasets. (b) Raw264.7 cells were treated with 50 ng/mL M-CSF and different concentrations of RANKL for six days. The gene expression of *IFIT1* was determined using qRT-PCR. (c) The protein expression of IFIT1 was determined using Western blotting analysis. (d) Raw264.7 cells were treated with 50 ng/mL M-CSF and 50 ng/mL RANKL for 0–12 days. The gene expression of *IFIT1* was determined using qRT-PCR. (e) The protein expression of IFIT1 was determined using Western blotting analysis. ns, no significance vs. control group or the indicated. *P < 0.05 vs. control group.

Bioinformatic analysis was performed to screen for differentially expressed genes in osteoarthritis. Six overlapping genes (*IFIT1, MMP1, CXCL10, WNT5A, RSAD2*, and *NDP*) were found to be upregulated in the GSE51588, GSE1919, and GSE75181 datasets (Figure 1(a)). *IFIT1* was selected for further investigation. The IFIT1 expression during OC formation was detected *in vitro*. Raw264.7 cells were differentiated into OCs by treatment with 50 ng/mL M-CSF and different concentrations of RANKL for six days. IFIT1 expression at the gene and protein levels, following stimulation with RANKL, was significantly increased in a dose-dependent manner (*P* < 0.05, Figure 1(b,c)). In addition, Raw264.7 cells were treated with 50 ng/mL M-CSF and 50 ng/mL RANKL for 0–12 days. IFIT1 expression, at the gene and protein levels was significantly increased by RANKL in a time-dependent manner (*P* < 0.05, Figure 1(d,e)). Ultimately, IFIT1 was highly expressed during OC formation. Raw264.7 cells treated with 50 ng/mL M-CSF and 50 ng/mL RANKL for six days to induce OC formation, were used in later experiments.

### Overexpression of IFIT1 accelerated OC formation induced by RANKL

**Figure 2.**
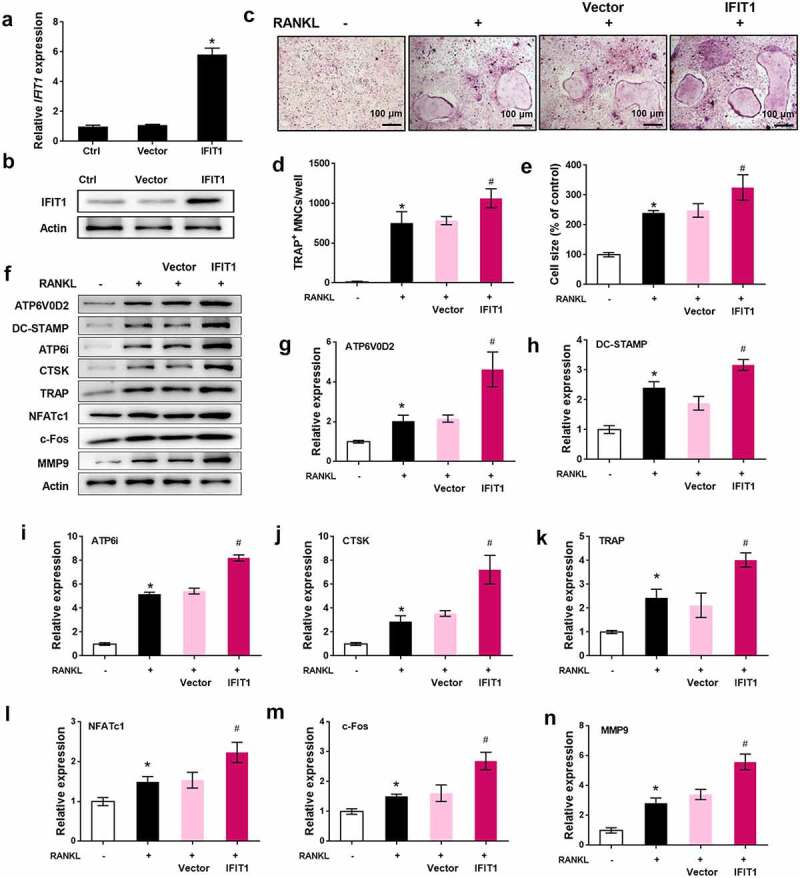
Overexpression of IFIT1 accelerated OC formation induced by RANKL. (a) Raw264.7 cells were transfected with PCMV6-IFIT1 or the empty vector. Expression level of *IFIT1* gene was determined using qRT-PCR. (b) Protein expression level of IFIT1 was determined using Western blotting analysis. (c) The transfected Raw264.7 cells were treated with 50 ng/mL M-CSF and 50 ng/mL RANKL for six days. The representative image of TRAP staining. (d) The number of multinucleate cell (≥ 3 nuclei) from TRAP staining. (e) Cell size from TRAP staining. (f) The representative image of Western blotting analysis. Quantitative levels of (g) ATP6V0D2, (h) DC-STAMP, (i) ATP6i, (j) CTSK, (k) TRAP, (l) NFATc1, (m) c-Fos, and (n) MMP9 proteins. *P < 0.05 vs. control group. #*P* < 0.05 vs. RANKL+Vector group.

To study the role of IFIT1 in OC formation, Raw264.7 cells were transfected with PCMV6-IFIT1. Transfection of Raw264.7 cells with PCMV6-IFIT1 significantly increased IFIT1 expression at both the gene and protein levels, indicating the efficiency of transfection (*P* < 0.05, Figure 2(a,b)). TRAP staining results showed that overexpression of IFIT1 significantly promoted the formation of OCs, as the number and size of multinuclear cells significantly increased (*P* < 0.05, Figure 2(c-e)). The effects of IFIT1 on OC-specific proteins were evaluated. As shown in Figure 2(f-n), levels of ATP6V0D2, DC-STAMP, ATP6i, CTSK, TRAP, NFATc1, c-Fos, and MMP9 were significantly increased in cells treated with RANKL (*P* < 0.05). Moreover, the levels of these proteins upregulated by RANKL were further elevated by IFIT1 overexpression (*P* < 0.05). These results suggest that IFIT1 accelerates the formation of OCs induced by RANKL.

### Silence of IFIT1 inhibited OC formation induced by RANKL

**Figure 3.**
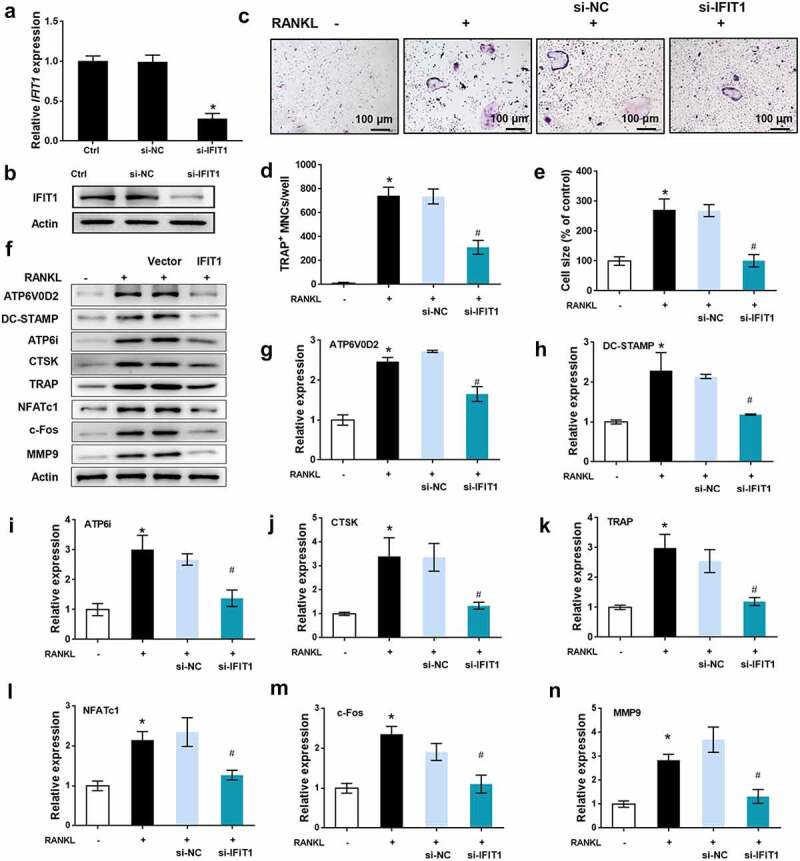
Silence of IFIT1 inhibited OC formation induced by RANKL. (a) Raw264.7 cells were transfected with si-IFIT1 or the negative control (si-NC). Expression level of *IFIT1* gene was determined using qRT-PCR. (b) Protein expression level of IFIT1 was determined using Western blotting analysis. (c) The transfected Raw264.7 cells were treated with 50 ng/mL M-CSF and 50 ng/mL RANKL for six days. The representative image of TRAP staining. (d) The number of multinucleate cell (≥ 3 nuclei) from TRAP staining. (e) Cell size from TRAP staining. (f) The representative image of Western blotting analysis. Quantitative levels of (g) ATP6V0D2, (h) DC-STAMP, (i) ATP6i, (j) CTSK, (k) TRAP, (l) NFATc1, (m) c-Fos, and (n) MMP9 proteins. *P < 0.05 vs. control group. #*P* < 0.05 vs. RANKL+si-NC group.

The role of IFIT1 in OC formation was further assessed by siRNA transfection. As illustrated in Figure 3(a,b), IFIT1 gene and protein levels were significantly reduced by si-IFIT1 transfection in comparison with the negative control (*P* < 0.05). TRAP staining results showed that the multinuclear cell number and size increased by RANKL were repressed by si-IFIT1 transfection (*P* < 0.05, Figure 3(c-e)). In addition, the levels of ATP6V0D2, DC-STAMP, ATP6i, CTSK, TRAP, NFATc1, c-Fos, and MMP9 upregulated by RANKL were decreased by si-IFIT1 transfection (*P* < 0.05, Figure 3(f-n)). Ultimately, silencing of IFIT1 inhibits the formation of OCs induced by RANKL.

### STAT3 was a downstream pathway of IFIT1

**Figure 4.**
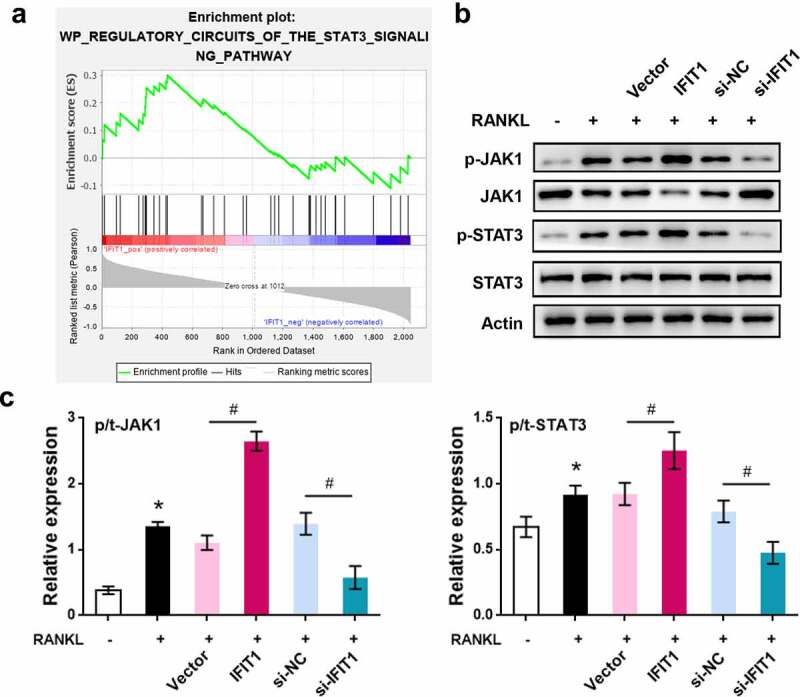
STAT3 was a downstream pathway of IFIT1. (a) STAT3 signaling pathway activated by IFIT1 was obtained by GSEA analysis. (b) Raw264.7 cells were transfected with PCMV6-IFIT1, si-IFIT1 or the negative controls. After transfection, cells were treated with 50 ng/mL M-CSF and 50 ng/mL RANKL for six days. Western blot was used to measure the expression of JAK1, STAT3 and the phosphorylated forms of the two proteins. (c) Fold change of phosphorylated JAK1 and STAT3 relative to total proteins. *P < 0.05 vs. control group. #*P* < 0.05 vs. the indicated group.

GSEA software was used to analyze the GSE1919 dataset, and pathways activated by IFIT1 were obtained. Considering the key role of STAT3 in OC formation and osteoarthritis progression, STAT3 signaling was selected for further investigation (Figure 4(a)). Western blotting results (Figure 4(b,c)) showed that overexpression of IFIT1 promoted the phosphorylation of JAK1 and STAT3 (*P* < 0.05). Conversely, IFIT1 silencing inhibited the phosphorylation of JAK1 and STAT3 (*P* < 0.05). These data confirmed that STAT3 is a downstream pathway for IFIT1, and IFIT1 contributes to the phosphorylation of JAK1 and STAT3.

### IFIT1 promoted OC formation by activating STAT3 signaling

**Figure 5.**
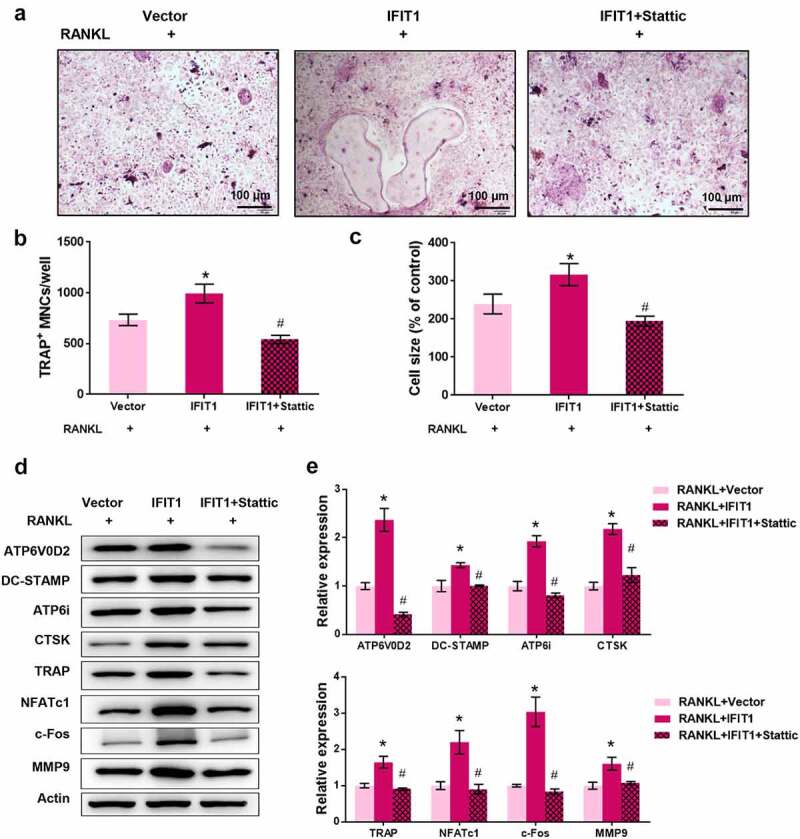
IFIT1 promoted OC formation by activating STAT3 signaling. (a) Raw264.7 cells were transfected with PCMV6-IFIT1 or the empty vector. After transfection, cells were treated with 50 ng/mL M-CSF and 50 ng/mL RANKL for six days. Stattic with a dose of 3 μM was used to treat cells for 24 h to inhibit STAT3 signaling. The representative image of TRAP staining. (b) The number of multinucleate cell (≥ 3 nuclei) from TRAP staining. (c) Cell size from TRAP staining. (d) The representative image of Western blotting. (e) Quantitative levels of ATP6V0D2, DC-STAMP, ATP6i, CTSK, TRAP, NFATc1, c-Fos, and MMP9 proteins obtained from Western blotting analysis. *P < 0.05 vs. RANKL+Vector group. #*P* < 0.05 vs. RANKL+IFIT1 group.

To validate the involvement of STAT3 signaling in IFIT1-mediated osteoclastogenesis, Raw264.7 cells were treated with 3 μM Stattic, a STAT3 inhibitor, for 24 h [25]. TRAP staining results showed that IFIT1 significantly increased the multinuclear cell number and size, but this effect was eliminated by Stattic (*P* < 0.05, Figure 5(a-c)). Furthermore, IFIT1 increased the levels of ATP6V0D2, DC-STAMP, ATP6i, CTSK, TRAP, NFATc1, c-Fos, and MMP9, but this effect was weakened by Stattic (*P* < 0.05, Figure 5(d,e)). Collectively, IFIT1 promotes OC formation by activating the STAT3 signaling pathway.

## Discussion

OCs play a critical role in mediating bone resorption. Additionally, OCs are involved in regulating hematopoiesis, bone formation, intraosseous angiogenesis, and osteocalcin hormone action [26–28]. Hyper bone resorption can cause bone degenerative diseases including osteoporosis and osteoarthritis, and bone resorption dysfunction can cause bone diseases such as sclerosis and compact osteogenesis imperfecta [29]. Drugs used for bone-related diseases mainly affect the process of bone resorption through the differentiation, function, and apoptosis of OCs. At present, multiple genes have been found to be abnormally expressed in OCs, providing potential pharmacological targets for bone resorption-related diseases. For example, MYC induces miR-320a expression to promote OC formation [7]. Methyltransferase 3 expression is increased during OC differentiation, and consequently regulates OC differentiation and function through different mechanisms [30]. Exploring more drug targets is conducive to drug research for bone resorption-related diseases.

IFIT1 is a well-known regulator of viral infection-induced immune response and inflammation [14]. However, the role of IFIT1 and its mechanism of action, in bone-related diseases, are poorly understood. In the present study, Raw264.7 cells were treated with M-CSF and RANKL to induce OC differentiation as described previously [31]. The gene and protein expression of IFIT1 was significantly increased by RANKL in Raw264.7 cells in a dose- and time-dependent manner. This, therefore, indicates the foundational involvement of IFIT1 in OC formation.

Furthermore, the *in vitro* results suggest that IFIT1 significantly promotes OC formation, and silencing of IFIT1 exerts the opposite effect. ATP6V0D2 and ATP6i are essential molecules that mediate OC formation and bone resorption [32,33]. TRAP is an iron-containing metalloenzyme that mediates OC resorption by degrading the endocytic bone matrix [34]. CTSK and MMP-9 degrade organic bone matrix-like type I collagen in bone tissue and increase bone resorption activity [35–37]. DC-STAMP is an essential cytokine in monocyte fusion and giant cell formation [8,38]. These factors are commonly used as biomarkers for bone metabolism and OC formation. In this study, IFIT1 positively regulated the expression of macrophage OC-related differentiation factors, promoting the fusion of single macrophages into multinucleated OC-like cells and improved OC function. These data suggest that IFIT1 may be a potential target to treat OC formation-related diseases. However, further studies are required, especially pit formation assay, to better understand the role of IFIT1 in bone resorption.

The STAT3 pathway has been reported to be involved in OC formation and differentiation [23,39]. It has been demonstrated that, peroxiredoxin II protects against lipopolysaccharide-induced OC formation through the inhibition of STAT3 [40]. Inhibition of Shc1-dependent STAT3 signaling suppressed OC formation and bone loss induced by oncostatin M [41]. In this study, the effect of IFIT1 on the STAT3 pathway was investigated. IFIT1 activated the STAT3 pathway by promoting the phosphorylation of JAK1 and STAT3. Furthermore, inhibition of STAT3 significantly reduced the effects of IFIT1 on multinucleated cells and OC formation-related proteins. These results suggest that IFIT1 promotes OC formation, at least partially, via regulation of STAT3 signaling. The production of RANKL has been recognized as a major output of STAT3 signaling [42,43]. It seems that IFIT1 acts as an accelerator in RANKL-induced OC formation. Specifically, RANKL induced the expression of IFIT1 in OC precursor cells, which further activated JAK1/STAT3 signaling to produce RANKL, ultimately leading to OC formation. To improve our understanding of OC formation, further studies are required to reveal other possible mechanisms used by IFIT1.

## Conclusion

The expression of IFIT1 was significantly increased during the differentiation of Raw264.7 cells into OCs induced by RANKL. Overexpression of IFIT1 promoted the formation of OCs and OC-specific proteins by regulating STAT3 signaling. The findings of this study provide a potential basis for further research and development of novel drugs for treatment of bone resorption diseases, such as osteoarthritis.

## Data Availability

The datasets used and/or analysed during the current study are available from the corresponding author on reasonable request.

## Highlights

1. IFIT1 is highly expressed in the event of osteoclastogenesis;
2. IFIT1 accelerates the formation of osteoclasts induced by RANKL;
3. Silence of IFIT1 controlled the osteoclast formation;
4. IFIT1 accelerates osteoclast formation by activating STAT3 signalling.
